# Multimodal Memory Components and Their Long-Term Dynamics Identified in Cortical Layers II/III but Not Layer V

**DOI:** 10.3389/fnint.2019.00054

**Published:** 2019-10-01

**Authors:** Dong Li, Guangyu Wang, Hong Xie, Yi Hu, Ji-Song Guan, Claus C. Hilgetag

**Affiliations:** ^1^Institute of Computational Neuroscience, University Medical Center Hamburg-Eppendorf, Hamburg, Germany; ^2^School of Life Science and Technology, ShanghaiTech University, Shanghai, China; ^3^School of Life Sciences, Tsinghua University, Beijing, China; ^4^Zhangjiang Laboratory, Shanghai Research Center for Brain Science and Brain-Inspired Intelligence, Institute of Brain-Intelligence Technology, Shanghai, China; ^5^Department of Health Sciences, Boston University, Boston, MA, United States

**Keywords:** cortical dynamics, cortical layers, multimodal learning and memory, 2-photon imaging, mice

## Abstract

Activity patterns of cerebral cortical regions represent the current environment in which animals receive multi-modal inputs. These patterns are also shaped by the history of activity that reflects learned information on past multimodal exposures. We studied the long-term dynamics of cortical activity patterns during the formation of multimodal memories by analyzing *in vivo* high-resolution 2-photon mouse brain imaging data of Immediate Early Gene (IEG) expression, resolved by cortical layers. Strikingly, in superficial layers II/III, the patterns showed similar dynamics across structurally and functionally distinct cortical areas and the consistency of dynamic patterns lasted for one to several days. By contrast, in deep layer V, the activity dynamics varied across different areas, and the current activities were sensitive to the previous activities at different time points, depending on the cortical locations, indicating that the information stored in the cortex at different time points was distributed across different cortical areas. These results suggest different roles of superficial and deep layer neurons in the long-term multimodal representation of the environment.

## Introduction

The brain can represent, integrate, and remember information from more than one sensory modality (Ghazanfar and Schroeder, 2006; Driver and Noesselt, 2008; Bruns and Röder, 2019; Leon et al., 2019; Taesler et al., 2019). This cross-modal integration is structured such that items can be represented both as a whole as well as a set of cross-modal details. In a complex environment, the learning of these integrated representations is a difficult task requiring repeated exposures to the multi-sensory stimuli. Moreover, the learning mechanisms need to address plasticity-stability trade-offs, by forming relevant new cross-modal associations while ignoring and forgetting irrelevant associations and preserving prior memories. As a result, the formation of cross-modal memories becomes a long-term dynamic process. Understanding the long-term dynamics of cortical memory representation in multimodal environments is not only a worthwhile topic by itself in brain research but also significant for inspiring the enhancement of cross-modal learning abilities of artificial brains (Parisi et al., 2019).

The cerebral cortex of the mammalian brain, which is parcellated into a multitude of structurally and functionally specific, layered areas, is believed to be involved in higher-order brain functions, including multisensory perception (Ghazanfar and Schroeder, 2006). Substantial evidence suggests that the cerebral cortex has both area-specific and layer-specific functions in the processes of learning and memory. For example, Phoka et al. (2016) found increased neural activity and concomitant ensemble firing patterns in mouse somatosensory cortex, specifically layers IV and Vb, sustained for more than 20 min after multi-whisker, spatiotemporally rich stimulation of the vibrissae. Kitamura et al. (2017) pointed out that contextual fear memory can be quickly produced at the onset of learning in the prefrontal cortex (PFC). Xie et al. (2014) discovered memory trace neurons in layers II/III of various areas of the mouse cortex. Wang et al. (2019) demonstrated that the cross-modal integration of visual and somatosensory inputs evoked specific neural responses in particular cortical areas, such as the primary visual (VISp) cortex and the retrosplenial cortex (RSC). Sellers et al. (2013, 2015) demonstrated that anesthetics could selectively alter spontaneous activity as a function of the cortical layer and suppress both multimodal interactions in the VISp cortex and sensory responses in the PFC. Despite these extensive observations, however, it remains unclear whether and how the long-term dynamics of cortical memory representations are cortical area- and layer-specific.

In this study, we investigated the long-term dynamics of cortical area- and layer-distributed cellular activity patterns during the formation of cross-modal memories by analyzing *in vivo* high-resolution 2-photon imaging data from BAC-EGR-1-EGFP mouse brains in multimodal environments. On each day, animals were put into one type of environment, receiving multimodal inputs. Several cortical locations from various brain regions of each subject were monitored, and within each location the neural activity patterns were represented by the firing rates of 6,000–15,000 neurons, across multiple cortical layers. During memory formation, the activity patterns of a particular day could be related to those on previous days, as analyzed using a prediction algorithm by a gradient boosting decision tree implemented in the LightGBM Python-package (Ke et al., 2017). We show that the long-term memory-related cortical dynamics are significantly layer-specific. In layers II/III, the dynamic patterns are similar across different types of cortical areas and different hemispheres, and the neural activities show an unspecific memory effect, that is, they are more sensitive to the recent history of one to several days than to activity of a longer time lapse, even if the more recent memories belong to different environments from the present one. In layer V, the activity patterns vary among cortical locations as the information stored in this laminar compartment at different previous time points appears distributed nonuniformly across different cortical areas. Those results, therefore, suggest different roles of superficial and deep layer neurons in the multimodal representation of the environment.

## Materials and Methods

### Animal Experiments

We analyzed data from four mice. The used mouse strain was BAC-EGR-1-EGFP (Tg(Egr1-EGFP)GO90Gsat/Mmucd from the Gensat project, distributed by Jackson Laboratories. Animal care was in accordance with the Institutional guidelines of Tsinghua University, and the entire experimental protocol was also approved by Tsinghua University. Imaging and data acquisition procedures were previously described by Xie et al. (2014). Specifically, mice were 3–5 months old, and received cranial window implantation; recording began 1 month later. To implant the cranial window, the animal was immobilized in custom-built stage-mounted ear bars and a nosepiece, similar to a stereotaxic apparatus. A 1.5 cm incision was made between the ears, and the scalp was reflected to expose the skull. One circular craniotomy (6–7 mm diameter) was made using a high-speed drill and a dissecting microscope for gross visualization. A glass-made coverslip was attached to the skull. For surgeries and observations, mice were anesthetized with 1.5% isoflurane. EGFP fluorescent intensity (FI) was imaged with an Olympus Fluoview 1200MPE with pre-chirp optics and a fast AOM mounted on an Olympus BX61WI upright microscope, coupled with a 2 mm working distance and a 25× water immersion lens (numerical aperture 1.05). The anesthetization was done 1 h after the animal explored a multisensory environment. Previous studies showed that, under these circumstances, anesthesia has very little effect on protein expression (Bunting et al., 2016) and that protein expression reflects the neural activities related to the environmental exploration very well (Xie et al., 2014).

We employed several types of environments for the animals. In principle, the environments were all multimodal environments, but of different complexity in terms of the sensory modalities. Home Cage was considered as the default, where, although the animals could see and touch the cage, as well as smell their own smells, they habituated to this environment and were closely familiar with the sensory inputs. Therefore, the visual, somatosensory and olfactory inputs in the Home Cage environment were all considered as weak, and this multimodal environment was considered as the simplest one compared to all others. An increased level of complexity was created by introducing stronger and specific inputs of certain modalities. To this end, we used another three boxes, labeled as contexts A, B, and C, which comprised different shapes, colors, materials of the floors, and combinations of different smells, so that animals received strong and specific visual, somatosensory, and olfactory inputs. In addition, we also employed strong light and sound stimuli in box C. When an animal was put into one of the boxes, it could experience three types of situations. Training A, B, or C meant that the animal received foot shocks that were strong enough to lead to freezing behavior, as part of conditioning for learning. At the same time, the foot shock could also be considered as a very strong and special somatosensory (nociceptive) input by itself. When the animal did not receive the foot shock, we labeled the boxes as Context A, B, or C if before training, or as Retrieval A, B, or C after training, respectively. In practice, the data used in this study do not include Context C or Retrieval C. Training C had the largest complexity in terms of sensory modalities when compared to the others, and interestingly, in the pre- and post-training phases, the animals displayed different behaviors, that is, freezing in Retrieval A, B, or C but not in Context A, B, or C (Xie et al., 2014), but we assumed that the provided sensory information was identical between the Context and Retrieval environments. Several other environments were also employed, which were more complex than the Home Cage, but simpler than those mentioned before. Enriched Environment and Tunnel were two boxes where animals could receive strong visual and somatosensory inputs. Another two simple environments were employed where the animals only received visual inputs of vertical or horizontal stripes.

**Figure 1:**
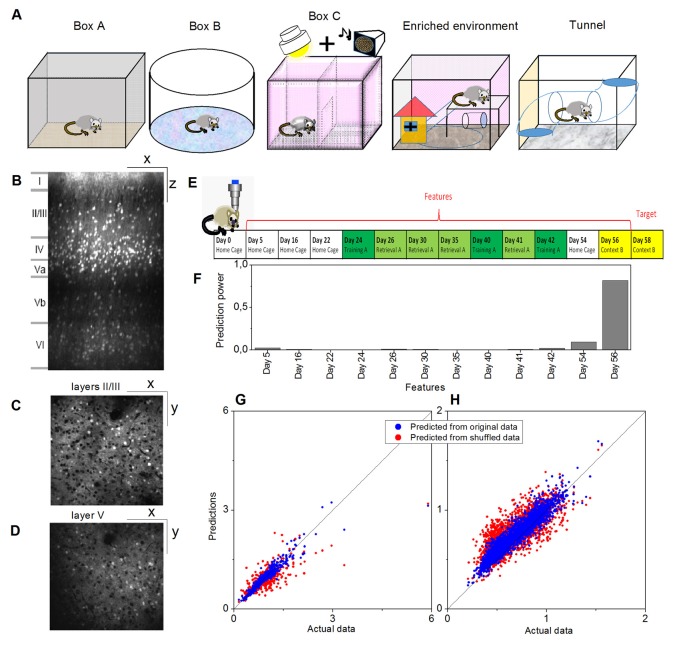
Example of a prediction of memory trace activities. **(A)** Illustration of different environments employed in this article. **(B)** One example slice of cortical location A1 (primary visual, VISp, left) of animal Ma and the manual annotations of the cortical layers, where x indicates the anterior-posterior direction and z indicates the superior-inferior direction. Panels **(C,D)** show slice examples of layers II/III and layer V (more specifically, Vb) of the same cortical location A1, where x indicates the anterior-posterior direction and y indicates the rostrocaudal direction. **(E)** The model was trained on layers II/III in this cortical location A1. Neural activities on Day 58 (context B) were used as the target, and the data for 12 previous days were used as the features. **(F)** Prediction powers of the 12 days in features. **(G)** Prediction performance of the model on other layers II/III neurons within the same location, and (H) prediction performance of the same model on all layer V neurons within the same location, where blue dots indicated the prediction from the original data [*R*^2^ = 0.82 in panel **(G)** and *R*^2^ = 0.81 in panel **(H)**] and red dots from the shuffled data [*R*^2^ = 0.61 in panel **(G)** and *R*^2^ = 0.48 in panel **(H)**].

Illustrations of the different environments are provided in Figure 1A and the sensory modalities encountered in the environments are summarized in Table 1. The respective environments that the four mice experienced are summarized in Table 2. The time of exploration in different environments varied from 5 min to 2 h, and the imaging was carried out about 1–1.5 h after the exploration, which was optimized to capture the neural activities of the animals in the explorations (Xie et al., 2014).

**Table 1:** List of the used multimodal environments.

Environments	Abbreviation	Visual^1^	Auditory^1^	Olfactory^1^	Somatosensory^1^
Home Cage	H	w	-	w	w
Context A	CA	S	-	S	S
Training A	TA	S	-	S	S + Footshock
Retrieval A	RA	S	-	S	S
Context B	CB	S	-	S	S
Training B	TB	S	-	S	S + Footshock
Retrieval B	RB	S	-	S	S
Training C	TC	S + Light	S	S	S + Footshock
Enriched Environment	EE	S	-	w	S
Tunnel	TU	S	-	w	S
Horizontal stimulus	HS	S	-	w	w
Vertical stimulus	VS	S	-	w	w

*^1^W means weak inputs, while S means strong, specific inputs*.

### Data Selection

For each mouse, 10–20 cortical locations were typically monitored, but we only selected the ones that could be scanned at least to a depth of layer Vb for all days of scanning. As a result, we selected 7, 8, 6, and 3 locations for those four mice, respectively, which covered motor, posterior parietal (PTLp), RSC, primary somatosensory (SSp), anterior medial visual (VISam), and VISp cortical areas on both the left and right hemispheres. The neuron positions in the images were automatically detected, as described in detail by Xie et al. (2014). If a neuron was missed in the detection for not more than 3 days, its missed activity values were filled as the median value of all the other neurons on that day. If, however, a neuron was missed in the detection for more than 3 days, the neuron would be excluded from the analysis. The area types and laminar compartments were manually annotated based on their cytoarchitecture by one expert (GW) and approved by all other experimental experts among the authors (HX, YH and J-SG). In practice, we first measured the relative position of each location with reference to the Bregma point and used the position to estimate the functional area type according to the atlas of the Allen Brain Institute (Lein et al., 2007; Oh et al., 2014). Subsequently, in the laminar compartment annotation, we mainly considered the depth, the neural density, and the morphology of the somata in terms of different sizes and shapes. In the functional area type annotation, we first discriminated motor/RSC from VISam/VISp/SSp/PTLp based on their distinct laminar structures and then further discriminated each area type based on their positions relative to the Bregma. Since the border between different functional regions is sometimes not very clear, some imaged locations are cross-functional regions, but these data were excluded from the analysis in this study. In this study, we focused our analysis on the activities in layers II/III and layer V. A summary of the data available for the analyzed four animals is provided in Table 2.

**Table 2:** Summary of the subjects.

Mouse	Scanned length (day)^1^	Scanned times	Environments	Selected Locations	Cortical areas covered^2^	Minimal number of neurons in layers II/III in each location	Maximal number of neurons in layers II/III in each location	Minimal number of neurons in layer V in each location	Maximal number of neurons in layer V in each location
Ma	131	32	Home cagel	7	PTLp (L)	3,683	6,158	311	2,626
			Training A		SSp (L)
			Retrieval A		VISp (L)
			Context B		RSC (R)
			Training C		VISp (R)
			Enriched Environment
			Tunne						
Mb	52	22	Home Cage	8	RSC (L)	2,041	4,863	399	1,104
			Training B		VISam (L)
			Retrieval B		VISp (L)
			Context A		Motor (R)
					RSC (R)
					VISam (R)
Mc	61	12	Home cage	6	PTLp (L)	842	3,775	116	986
			Training A		RSC (L)
			Retrieval A		VISam (L)
			Context B		PTLp (R)
					VISam (R)				
Md	55	26	Home cage	3	Motor (R)	2,821	5,492	342	1,400
			Horizontal stimulus		SSp (R)
			Vertical stimulus
			Enriched Environment

*^1^Calculated from the first to the last day of scanning, and signed as Day 0, Day 1, etc. ^2^L and R mean the left and right hemispheres, respectively*.

### LightGBM Prediction Approach

We analyzed the long-term dynamics of cortical memory representations as a regression problem, by predicting the activity pattern on a certain day based on the history of activity patterns. Practically, we used the gradient boosting decision tree implemented in the LightGBM (Ke et al., 2017) Python-package.

For each prediction, we needed to select training, validation, and test data. Once the activities on a certain day were selected as the target, their values in the training and validation data sets were used as the labels. The values in the test data were not used in the prediction process but were used as ground truth to evaluate the prediction performance. Features included the activities on the previous days. The parameters used in the LightGBM prediction are shown in Table 3.

**Table 3:** Parameters for LightGBM prediction.

Num_leaves	Objective	Min_data_in_leaf	Learning_r_ate	Feature_fraction	Bagging_fraction	Bagging_freq	Metric	Num_threads
10	Regression	1	0.05	0.93	0.93	1	l2	4

Since we used “l2” for the parameter “metric” in the evaluation process (which means that the mean square error was the target to be optimized in the process of the regression), we calculated the mean square error δ between the prediction results and the ground truth as an accuracy estimate. To generate controls, we shuffled the data on the feature days for each neuron.

### Cross-location Prediction

For each animal, we selected one specific laminar compartment Λ (Λ was either in layers II/III or layer V). One model was trained by using the training and validation data from one cortical location i_Λ_, and predictions were subsequently performed by using the test data from a different location j_Λ_ in the same laminar compartment. At this stage, the target was always selected as the data on the last day when the animal’s brain was scanned, and the features were the data on all the previous days that were available, excluding Day 0, in total from 10 to 30 days (see Table 4). To make all pairs of predictions comparable, at this stage, for each animal we needed to select the data sizes of training, validation and test data, respectively, so as to have identical data for every model. To this end, for each mouse, we first identified the minimal number of neurons in every location in layers II/III or layer V in the data set, which turned out to be 311, 399, 116, and 342 neurons, respectively (Table 4). This number was the size of the test data for each mouse, and the size of training and validation data were 90% and 10% of these numbers, respectively, as seen in Table 4. With those fixed numbers, the data sampling was random, and the validation and the training data sets never had overlaps.

**Table 4:** Summary of cross-location prediction.

Mouse	Number of days in features	Data points in training/validation/test set	Total pairs of comparison	Number of worse performance (significant ones)^1^	Number of better performance (significant ones)^1^	Worst difference in performance^1^	Best difference in performance^1^
Ma	30	297/33/311	42	40 (18)	2 (2)	−0.377	0.124
Mb	20	359/39/399	56	38 (25)	18 (5)	−0.362	0.234
Mc	10	104/11/116	30	20 (7)	10 (4)	−0.198	0.255
Md	24	307/34/342	6	6 (4)	0 (0)	−0.504	−0.121
sum	-	-	134	104 (54)	30 (11)	-	-

*^1^Compared the performance in layer V to layers II/III*.

To evaluate the prediction results, we not only calculated the square error δ(i_Λ_, j_Λ_), but also shuffled the data on the feature days in the test data sets 20 times for the comparison of each pair of training-test locations, and predicted the target each time, so as to obtain another 20 predicted results. The control square error δ_s_(i_Λ_, j_Λ_) was calculated by using the average of the 20 predicted results from the shuffled data. The relative error was then calculated as δ_r_(i_Λ_, j_Λ_) = δ(i_Λ_, j_Λ_)/δ_s_(i_Λ_, j_Λ_). We defined the prediction quality measurement κ as κ(i_Λ_, j_Λ_) = exp[−δ_r_(i_Λ_, j_Λ_)], and the matrix M_κΛ_, whose off-diagonal entry at the ith row and jth columns was κ(i_Λ_, j_Λ_) and diagonal elements were all empty. M_κΛ_ was, therefore, able to reflect how the memory-dependent dynamics of the neural populations from the testing location were similar to the training location.

We repeated these predictions and evaluations 10 times so as to obtain 10 M_κΛ_. The differences in prediction performances for layers V and II/III could be demonstrated in two ways. In the first instance, we averaged all the 10 M_κΛ_ for each layer compartment, to obtain ${\tilde{M}}_{\kappa \Lambda }$, and calculated ${\tilde{M}}_{\kappa }={\tilde{M}}_{\kappa \text{II/III}}-{\tilde{M}}_{\kappa \text{V}}$, and finally used the matrix M_s_ = (M_κ_+ M_κ_^T^)/2 to demonstrate the difference. If one entry was 0, it meant that the predictions for layer V and layers II/III had the same performance in the corresponding pair of locations, and the values larger (or smaller) than the 0 mean prediction in layers II/III (or layer V) performed better. In the second instance, we directly compared the difference of the 10 values between κ(i_V_, j_V_) and κ(i_II/III_, j_II/III_) to search for the significant difference (*p* < 0.01, *t*-test with Bonferroni correction).

### Intra-location Prediction

Intra-location prediction was basically performed in the same way as cross-location prediction. The only difference was that, since the test data set came from the same population as the training and validation data sets, it was necessary to make sure that those data did not overlap. To this end, we divided the data equally across each location and laminar compartment into 10 groups. For each prediction, we sampled one group of neurons, and randomly sampled 50 neurons from this group as the validation data, sampled another two groups of neurons, and randomly sampled 100 neurons from these two groups as the test data, and randomly sampled 350 neurons from the left groups as the training data. Locations whose layers II/III or layer V did not contain at least 500 neurons were excluded from this part of analysis.

### Prediction Power

Once a model Mod^(r)^ was trained, and we signed the set of feature days as S^(r)^, LightGBM returned the total gains of splits for each feature G_D_^(r)^, where D indicates the feature day used in this model. We therefore directly used the gain normalized by their summation, i.e., $P_{\text{D}}^{\left(\text{r}\right)}=G_{\text{D}}^{\text{(r)}}/\sum_{D\in S_{\left(\text{r}\right)}}G_{\text{D}}^{\left(\text{r}\right)}$ to indicate the prediction power of the feature day D in model Mod^(r)^. Because the prediction power was the property within a model itself, insensitive to its performance with test data, and the measurement was a value normalized within the model, for each model we used a large part (90%) of the neurons within the population (80% as training data and 10% as validation data).

### Repeat Environment Prediction

In this part of the study, we used four features to predict the target activities. Day 0 was always excluded from the analysis, and the data of the next three scanning time points (labeled as Day S_1_, Day S_2_, and Day S_3_) were always included in the features, in order to generate controls to evaluate the prediction performance. However, in order to eliminate the predictive effects from those 3 days that could be different among the situations which we were going to compare, we shuffled the neurons on each of those 3 days. For each animal, from Day S_4_ onwards, we looked for the next scanning day on which the mouse was put into a repeated environment for the first time, and included this pair of repeated environments into the analysis, except for that between those days, when the mouse used to be put into the same box, even though the environment was different. For instance, in the sequence consisting of Retrieval A (Day S_n_), Home Cage (Day S_n + 1_), and Retrieval A (Day S_n + 2_), the pair of Day S_n_ and Day S_n + 2_, which has the environment-repeat interval *Inv* = S_n + 2_-S_n_, would be included in the analysis, but in the sequence consisting of Retrieval A (Day S_n_), Training A (Day S_n + 1_), and Retrieval A (Day S_n + 2_), the pair of Day S_n_ and Day S_n + 2_ would be excluded. For each selected pair, we used the data of the previous day together with the aforementioned shuffled data on Day S_1_, Day S_2_, and Day S_3_ to predict the activities on the following day, which resulted in a mean square error δ(i_Λ_) in location (i_Λ_ in layer compartment Λ), and we shuffled the days in the test data, resulting in δ_s_(i_Λ_). Therefore, we eventually obtained δ_r_(i_Λ_) = δ(i_Λ_,)/δ_s_(i_Λ_), which measured the performance of this prediction, where smaller δ_r_(i_Λ_) indicates better prediction. We repeated the prediction 100 times within each location i_Λ_, and obtained the averaged value <δ_r_(i_Λ_)>, where <·> stands for the average over trials. Within each location i_Λ_, we still randomly divided the neurons into 10 groups, and for each prediction, we randomly selected four groups (40% of the data) as the training data, one group (10% of the data) as the validation data, and left the other five groups (50% of the data) as the test data.

We calculated the average of <δ_r_(i_Λ_)> among all the locations of the mouse, to obtain the mean value ${\bar{\delta }}_{r}$ and the standard deviation σ(δ_r_) so that we could analyze their dependence on the environment-repeat interval *Inv*, simply by using lining fitting ${\bar{\delta }}_{r}=\rho_{m}\cdot Inv+\alpha_{m}$ and σ(δ_r_) = ρ_s_ · *Inv* + α_s_, respectively. To analyze their dependence on the multimodal environments, we selected the two most often repeated environments for each mouse (eventually 5–8 repeating times), and compared ${\bar{\delta }}_{r}$ and σ(δ_r_) twice over all the repeats between those two environments. First, we made the comparison by using the original values, and afterward, in order to eliminate the influence of the different environment-repeat interval *Inv* as much as possible, we made the comparison again by using a kind of modified values, which equalled the original values minus *Inv* times the fitted slopes, namely ${{\bar{\delta }}^{\prime }}_{r}={\bar{\delta }}_{r}-\rho_{m}\cdot Inv$, and $\sigma^{\prime }(\delta_{r})=\sigma (\delta_{r})-\rho_{s}\cdot Inv$, respectively.

Subject Mc was excluded from this part of the analysis because it only experienced very few environment repeats.

### Supplementary Explanations of the Terminology Used in This Study

In this section, we provide supplementary explanations of the terminology used for various purposes in this study, in order to avoid misunderstandings of the terms.

- *Type of cortical area* and *cortical location*: there are two concepts regarding the cortical imaging positions that may be potentially confused. Therefore, we used two distinct terms to distinguish them. *Type of cortical area* means the structural-functional cortical area, for example, VISp, VISam, motor, etc, whereas *cortical location* means one of several particular positions that was monitored in the research. To label the cortical locations of a mouse Mx (x stands for a, b, c, d), we used numbers following the capital form of x (for example, for mouse Ma, those cortical locations were labeled A1, A2, A3, etc). Different cortical locations might, therefore, belong to the same type of cortical area.
- *Environment* and *Environment repeat*: in this work, *environment* describes the set of all the environmental conditions that could be perceived by any sensory modality, for example, the box or cage in which the animal was located, with particular walls, floors and even toys, the smells, the sounds, the foot-shocks, and any other external stimuli. *Environment repeat* means an animal experienced the same environment for another time.
- *Complexity*: in this study, the complexity of the environment comprises the range, types, and strength of the stimuli provided in the different sensory modalities.
- *Model*: throughout this article, *model* is only used in the sense of a machine learning model, and never refers to an animal model or any other kind of model.

## Results

### Predictability

**Figure 2:**
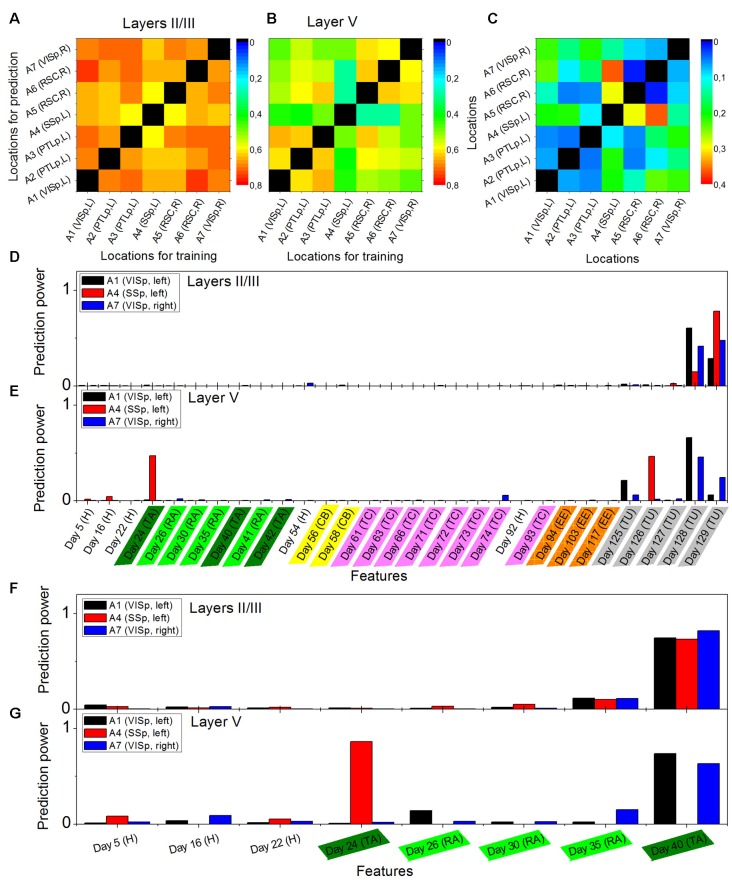
Cross-location prediction performance ${\tilde{M}}_{\kappa \Lambda }$ in layers II/III **(A)** and in layer V **(B)**, and their relative difference M_s_ **(C)**, by using the data from animal Ma. Diagonal elements do not have values. Prediction power distributions of layer II/III model **(D)** and layer V model **(E)** trained in cortical location A1 (left VISp), A4 (left primary somatosensory, SSp, and A7 (right VISp), when the neural activities on Day 130 (Tunnel) was used as the target and all previous days in the data set as the features. Panels **(F,G)** show the prediction power distributions of layer II/III model and layer V model, respectively, when the neural activities on Day 41 (Retrieval A) was used as the target and eight previous days in the data set as the features. Abbreviations: H, home cage; TA, training A; RA, retrieval A; CB, context B; TC, training C; EE, enriched environment and TU, tunnel.

Activities of cortical neurons could be predicted by using a gradient-boosting decision tree, taking their past activities as features and already knowing some of the activities at the target day as the training labels. One example is shown in Figure 1, where a model was trained on layers II/III in a cortical location of mouse Ma, and the neural activities on Day 58 (context B) were used as the target, and the data on twelve previous days were used as the features (Figures 1E,F). The prediction from this model by using the original data produced much more similar results to the actual data than by using shuffled data (Figure 1G, where *R*^2^ = 0.82 for original data vs. *R*^2^ = 0.61 for shuffled data). Moreover, although prediction performance varied, a model trained in a laminar compartment of a cortical location was able to predict the neural activities in a different laminar compartment (Figure 1H) or in a different cortical location (Figures 2A–C).

### Cross-location and Intra-location Predictions

The performance of cross-location prediction was significantly layer-specific. In layers II/III, any model trained from one cortical location could well predict the neural activities in other cortical locations, whether they belonged to the same type of cortical area or the same hemisphere (Figure 2A). By comparison, cross-location prediction performed much worse for layer V (Figures 2B,C and Table 4). Specifically, when we compared the different prediction performances in layer V to layers II/III of each pair of training-test locations, for all four animals among all the 134 pairs, we obtained 104 worse performances in layer V compared to layers II/III (in terms of the averaged value κ), out of which 54 were significant (*p* < 0.01, *t*-test with Bonferroni correction within each animal), whereas we had only 30 better performances in layer V, out of which only 11 were significant (Table 4).

Intra-location prediction showed the same bias, that is, it performed worse in layer V than in layers II/III, but the difference was much less significant than cross-location prediction (when Table 5 is compared to Table 4). Specifically, among all the 17 comparisons, there was only one result showing significant difference.

**Table 5:** Summary of intra-location prediction.

Mouse	Number of days in features	Data points in training/validation/test set	Total pairs of comparison	Number of worse performance (significant ones)^1^	Number of better performance (significant ones)^1^	Worst difference in performance^1^	Best difference in performance^1^
Ma	30	350/50/100	5	2 (0)	3 (0)	−0.346	0.035
Mb	20	350/50/100	6	4 (0)	2 (0)	−0.173	0.151
Mc	10	350/50/100	4	2 (0)	2 (0)	−0.102	0.290
Md	24	350/50/100	2	1 (1)	1 (0)	−0.265	0.006
sum	-	-	17	9 (1)	8 (0)	-	-

*^1^Compared the performance in layer V to layers II/III*.

Furthermore, we found that in the cross-location prediction, the large differences in performance tended to appear for pairs of locations involving different types of cortical areas (see for example locations A1 and A4 in Figure 2C, which were in left VISp and left SSp, respectively) or different hemispheres (see for example locations A1 and A7 in Figure 2C, which were in left VISp and right VISp, respectively).

The analysis of the prediction powers of the days in history helped us obtain deeper insights into the differential performances of layer V and layers II/III predictions. Taking the models trained on A1 (left VISp), A4 (left SSp), and A7 (right VISp), for example, the distributions of the prediction powers for the models in layers II/III were very similar (Figure 2D). Specifically, most powerful predictors were those on the most recent days (such as Day 128 and Day 129 when the targets were on Day 130). In layer V, the prediction power had significantly different distributions for the models trained on those three locations (Figure 2E), where for A1 and A7, 3 days (Day 125, Day 128 and Day 129) with the same environment as the target day (Tunnel) had high prediction powers and only for A7, 1 day (Day 74) also had high prediction power, whereas for A4, 2 days (Day 24, Training A and Day 126, Tunnel) had significantly high prediction powers. Even if we used the data within a short duration in those three locations to train models, for example, Day 41 (Retrieval A) as the target day and all previous days as features, we can still find those different patterns of the prediction power distributions between layers II/III and layer V. In layers II/III, the distributions were still very similar (Figure 2F), but in layer V, the distributions were widely different (Figure 2G).

### Repeat Environment Prediction

**Figure 3:**
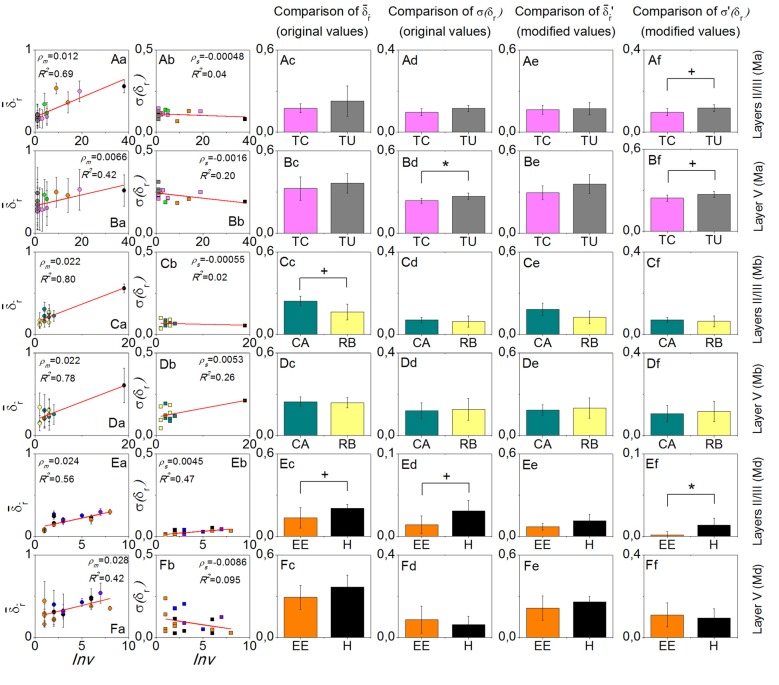
Results of the environment-repeat prediction. Rows from top to bottom are layers II/III of mouse Ma, layer V of mouse Ma, layers II/III of mouse Mb, layer V of mouse Mb, layers II/III of mouse Md, and layer V of mouse Md. The first column is the dependence of $\bar{\delta }r$ on the environment-repeat interval Inv, where error bars in fact indicate the standard deviation σ(δr), and the red lines are the linear fitting results. The second column shows the dependence of σ(δr) on Inv. The third to the sixth columns show the comparisons of $\bar{\delta }r$ and σ(δr) with the original and modified data, respectively, between the most often repeated environments that each mouse experienced. Colors in the figure are used to discriminate environment types. ^+^*p* < 0.1 and *p < 0.05.

For each mouse, $\bar{\delta }r$ in layers II/III was always more sensitive to environment-repeat interval *Inv* compared to layer V, reflected by the bigger slopes ρ_m_, or bigger *R*^2^ values of the line fitting results, or both (the first column of Figure 3). σ(δr) did not have a very strong interrelation with *Inv* (the second column of Figure 3). In the comparisons of $\bar{\delta }r$ and σ(δr) with the original values between the most often repeated environments, we only found one result which had statistical significance (*p* < 0.05), which was the σ(δr) in layer V of mouse Ma between Training C and Tunnel. After modifying the values, the significance did not change too much (*p* is still smaller than 0.1). Other comparisons that had small *p* values (<0.1) included $\bar{\delta }r$ in layers II/III of mouse Mb between Context A and Retrieval B (*p* > 0.1 after the modification), σ(δr) on layers II/III of mouse Md between Enriched Environment and Home Cage (*p* > 0.1 after the modification), and σ(δr) on layers II/III of mouse Md between Enriched Environment and Home Cage (*p* < 0.05 after the modification). In addition, σ(δr) in layers II/III of mouse Ma between Training C and Tunnel did not have a small *p*-value (*p* > 0.1), but it became smaller than 0.1 after modification.

## Discussion

### Interpretation of the Predictions

Although cortical activity patterns in the context of learning and memory appear very complex, they are not purely random. Rather, they are sensitive to outside stimuli as well as their own histories (Soon et al., 2008). The prediction approach employed in this study indeed followed such a hypothesis, that cortical neurons can represent long-term memories in multimodal environments, so as to have long-term memory-dependent dynamics. If a model trained within one neural population can also successfully predict the neural activities in another population, it means that within the considered history period, these two populations have similar memory-dependent dynamics. Moreover, the features with high prediction powers indicate the time point when the fresh information in the history that is useful for forming the current activity patterns starts to encode in the neural populations. However, the days of the features with very low prediction powers do not necessarily mean that their activities do not correlate with the activities on the target day. Another possibility may be that they do not encode additional useful information for predicting the neural activities on the target day, on top of the days of higher prediction powers.

As a result, we show that within the same cortical location and same laminar compartment, neurons indeed have similar long-term memory-dependent dynamics. Even across layers, or across areas, the neurons may still have certain similarities in these long-term memory-dependent dynamics, but the similarities vary from case to case.

### Comparison Between Layers II/III and Layer V

Many parts of the neocortex are involved in learning and memory processes (McClelland et al., 1995). In this study, while aiming to explore the layer-specific long-term memory-dependent dynamics of cortical neural activities, we specifically selected layers II/III and layer V for a number of reasons. In particular, both layers II/III and deep cortical layers have been shown to play important roles in learning and memory in previous studies (Xie et al., 2014; Hayashi-Takagi et al., 2015; Gao et al., 2018; Wang et al., 2019) and the quality of the data under study is good in multiple locations scanned down to layer V (more specifically, to layer Vb). Thus, layers II, III, Va and Vb turned out to be the good candidates for this study. Ideally, we would have liked to study all these laminar compartments individually, but in practice, the approach was subject to some restrictions.

First, layer II and layer III are not easy to discriminate based on their cytoarchitecture as obtained in the protein expression data set (Li et al., 2019); thus, we had to analyze them as one joint laminar compartment. There may be some differences in terms of the long-term memory-dependent dynamics between these layers, but we have to leave this problem to future studies.

Likewise, layer Va cannot be analyzed individually because it is too thin and difficult to discriminate in the data set. In some locations, the boundaries between layers Va and IV or the boundaries between layers Va and Vb are vague. The thickness fluctuations are already larger than the thickness of layer Va itself. Analyses of the data from the individual layer compartment Va in this research would, therefore, comprise too much noise. Combining layers Va and Vb into a single laminar compartment layer V appeared, therefore, to be the best solution. However, it is worth mentioning here that, since layer Vb contains many more neurons than layer Va, the properties of layer V that we revealed in this work may in fact mainly reflect the properties of layer Vb. In line with this conclusion, results are qualitatively the same when we used data just for layer Vb instead of joint layer V (see the Supplementary Material). We acknowledge that in previous studies the response properties to external stimuli in layer Va was significantly different from layer Vb (de Kock et al., 2007), but due to the described technical limitations, the potential difference in the long-term memory-dependent dynamics of these laminar subcompartments has to be left as an open problem for future research.

In any case, the comparison between cross-location predictions in layers II/III and V already revealed differences between superficial and deep layer cortical neural activities in the long-term memory-dependent dynamics. These differences are not due to the relatively different data qualities at different scanning depths, as we show in the intra-location prediction that the difference between these two-layer compartments is much less significant.

In layers II/III, the prediction performances are always quite good in any pair of training-test locations (in the example shown in Figures 2A–C, and mainly distribute between 0.6 and 0.8; in comparison, in the intra-location prediction in layers II/III of this mouse, all approximate to 0.8, although technically they are not comparable due to the different sizes of training, validation and test data sets). This means that in layers II/III, the cortical memory representations have very similar long-term dynamics across cortical areas. This result is not equal to, but matches, the results of previous studies that memory trace neurons were found in layers II/III, irrespective of the cortical areas (Xie et al., 2014). In the present study, however, we did not specifically focus on memory trace cells, but the whole pattern of neural activities. Further analysis revealed that the neural activity patterns in layers II/III are always sensitive to the very recent activities in history, which implies ongoing dynamics in layers II/III with a time scale of one to several days. The functional role of these dynamics in learning and memory processes need to be investigated in future research.

In contrast, in layer V, cross-location predictions perform much worse (in the example shown in Figures 2A–C, some $\tilde{\kappa }$(i_II/III,j_II/III) can be as low as 0.2; in comparison, in the intra-location prediction in layer V of this mouse, most of $\tilde{\kappa }$(i_II/III,j_II/III) also approximate to 0.8, although, again, they cannot technically be comparable due to the different sizes of data sets), but between the locations that belong to the same types of cortical areas and same hemispheres, the performances are not too bad, which already implies the different functional roles of cortical areas in layer V in long-term learning and memory processes. Consistently, the cross-location predictions within the associative cortices, including PTLp and RSC (dorsal) show a much more similar performance between layers II/III and layer V, whereas the sensory cortices, including visual cortex and somatosensory cortex, show larger difference between those two-layer compartments. Results from a comparison between the different prediction power distributions further indicate that information encoded in the neural activities that is useful for the neural responses to the current environment is segregated and stored in layer V in different cortical locations. In other words, when the animal is located in a particular environment, its layer V neurons form the patterns as a result of both the response to external multi-modal inputs and the retrieval of previously stored information of different modalities, where the information stored at different previous time points is distributed across different cortical areas. However, one should mention that our approach used in this work did not enable us to localize the cortical areas for any particular feature of information, which will be an important task in future studies. In addition, the anatomical mechanisms underlying the layer-specific long-term dynamics of the neural activities are also an intriguing topic that needs to be investigated in future studies.

### Repeat Environment Prediction

At the current stage, we could reasonably hypothesize that neural activities in layers II/III are more sensitive to temporal information, but relatively more insensitive to the complexity in terms of the sensory modality of the environments compared to layer V, whereas, when the environment becomes more complex, neural activities in layer V coordinate more strongly across cortical areas to represent the environment. This hypothesis motivated us to test the repeat environment prediction.

Since δ_r_(i_Λ_) measures in location i_Λ_ how well the present neural activities can predict the activities in a repeated environmental exposure in the future, it basically reflects how reliably an environment-specific cortical pattern can be reactivated. Therefore, the variable $\bar{\delta }r$ reflects the overall reliability of a layer compartment for reactivating the environment-specific cortical patterns, and σ(δr) reflects the differences of these reliabilities across cortical locations/areas.

The results show that $\bar{\delta }r$ is sensitive to the environment-repeat interval *Inv*, which is consistent with a decay process of memory. In comparison, for layers II/III, $\bar{\delta }r$, is more sensitive to *Inv* than layer V, which verifies the first part of our hypothesis that layers II/III is more sensitive to temporal aspects of representing information than layer V.

Among all three pairs of environments that we compared, only Training C comprised more sensory modalities than Tunnel, so we expected that σ(δr) would be smaller in Training C than Tunnel in layer V, which turned out to be true (Figures 3Bd,Bf). This result, therefore, verifies the second part of our hypothesis, that layer V is more sensitive to the complexity of remembered contexts in terms of sensory modalities.

Even more interestingly, we know that in Context A and Retrieval B, the animal had significantly different behaviors, that is, it showed freezing in Retrieval B but not in Context A (Xie et al., 2014), but the environments Context A and Retrieval B comprise the same sensory modalities. In comparison, their σ(δr) in layer V or layers II/III did not show a significant difference (Figures 3Cd,Cf,Dd,Df). Therefore, the difference in the behaviors was not related to the same aspect of the cortical activities which relates to the sensory modalities of the environments. The only difference of $\bar{\delta }r$ in layers II/III was in fact due to the different environment-repeat intervals (compared Figure 3Cc to Figure 3Ce).

The data of animal Md gave some unexpected results (the last two rows of Figure 3), but since the studied cortical locations of this animal were limited (only three locations from two cortical areas), it is difficult to interpret them in a convincing way.

### Regarding the Methodology

LightGBM is a machine-learning package based on decision trees. Therefore, its prediction ability is derived from the correlations between the target and the features, given that the data are cut into leaves. Similar results could potentially be achieved by correlating the activities on different days. Given the massive number of data points, it is also possible that some deep learning methods might give better prediction results than LightGBM. However, a higher prediction accuracy was not our goal in this work, and deep learning methods usually cannot reveal the deeper mechanisms underlying the different dynamics, as revealed here, based on the prediction power distributions.

## Conclusion

Activities of cortical neurons are sensitive to both the current environment in which the animals receive stimuli from various modalities as well as the history of activities reflecting the learned experience of various types of environments, forming long-term memory-dependent activation dynamics. These long-term dynamics are specific for different cortical layers. In layers II/III, they are similar across different cortical areas and different hemispheres, implying a distributed cortical memory system in layers II/III that integrates multisensory information into the memory. The layers II/III memory network shows ongoing dynamics with a time scale of one to several days. In layer V, such consistent memory signal dynamics across-time are lost and their patterns are varied among cortical locations. Between the locations that belong to different types of cortical areas, or belong to different hemispheres, the differences between the long-term memory-dependent dynamics tend to be bigger. Thus, information that has been stored at different previous time points is distributed across layer V of different cortical areas, which determines the present activity patterns, jointly with the current multimodal inputs from the environment. Different roles of superficial and deep layers neurons in cross-modal learning process are, therefore, suggested by the layer-specific long-term dynamics of cortical memory representations.

## Data Availability Statement

The raw data supporting the conclusions of this manuscript will be made available by the authors, without undue reservation, to any qualified researcher.

## Ethics Statement

The animal study was reviewed and approved by Institutional Animal Care and Use Committee, Tsinghua University.

## Author Contributions

J-SG and CH designed the research. GW, HX and YH worked on the experiments and collected the data. DL and GW analyzed the data. DL, J-SG and CH wrote the article. All authors approved the article.

## Conflict of Interest

The authors declare that the research was conducted in the absence of any commercial or financial relationships that could be construed as a potential conflict of interest. The reviewer MG-C declared a shared affiliation, though no other collaboration, with one of the authors CH to the handling Editor.
