# Supplementary material for: Multimodal Memory Components and Their Long-Term Dynamics Identified in Cortical Layers II/III but Not Layer V
Source: Front Integr Neurosci. 2019 Oct 1;13:54. doi: 10.3389/fnint.2019.00054 (PMC6779795; doi:10.3389/fnint.2019.00054)
Supplement: Supplementary file 1 [file Data_Sheet_1.PDF]

Supplementary Figures

(Analysis by using layer Vb instead of layer V)

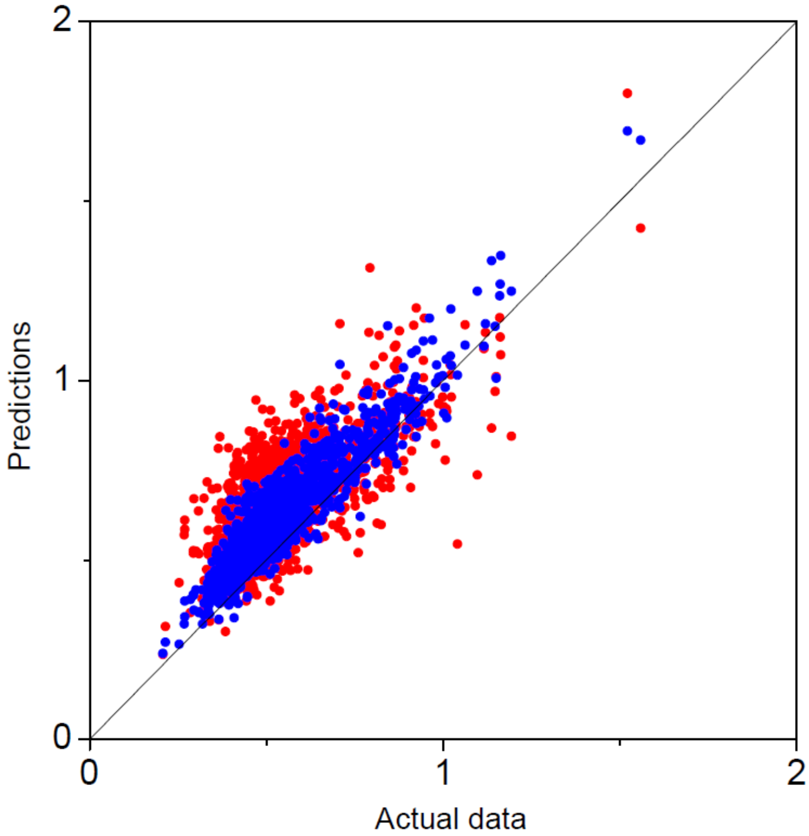

Figure S1 Prediction performance of the model trained in layers II/III neurons applied on all layer Vb neurons within the same location A1 of mouse Ma, where blue dots indicated the prediction from the original data and red dots from the shuffled data. This figure shows the result of layer Vb corresponding to that of layer V in Figure 1 H.

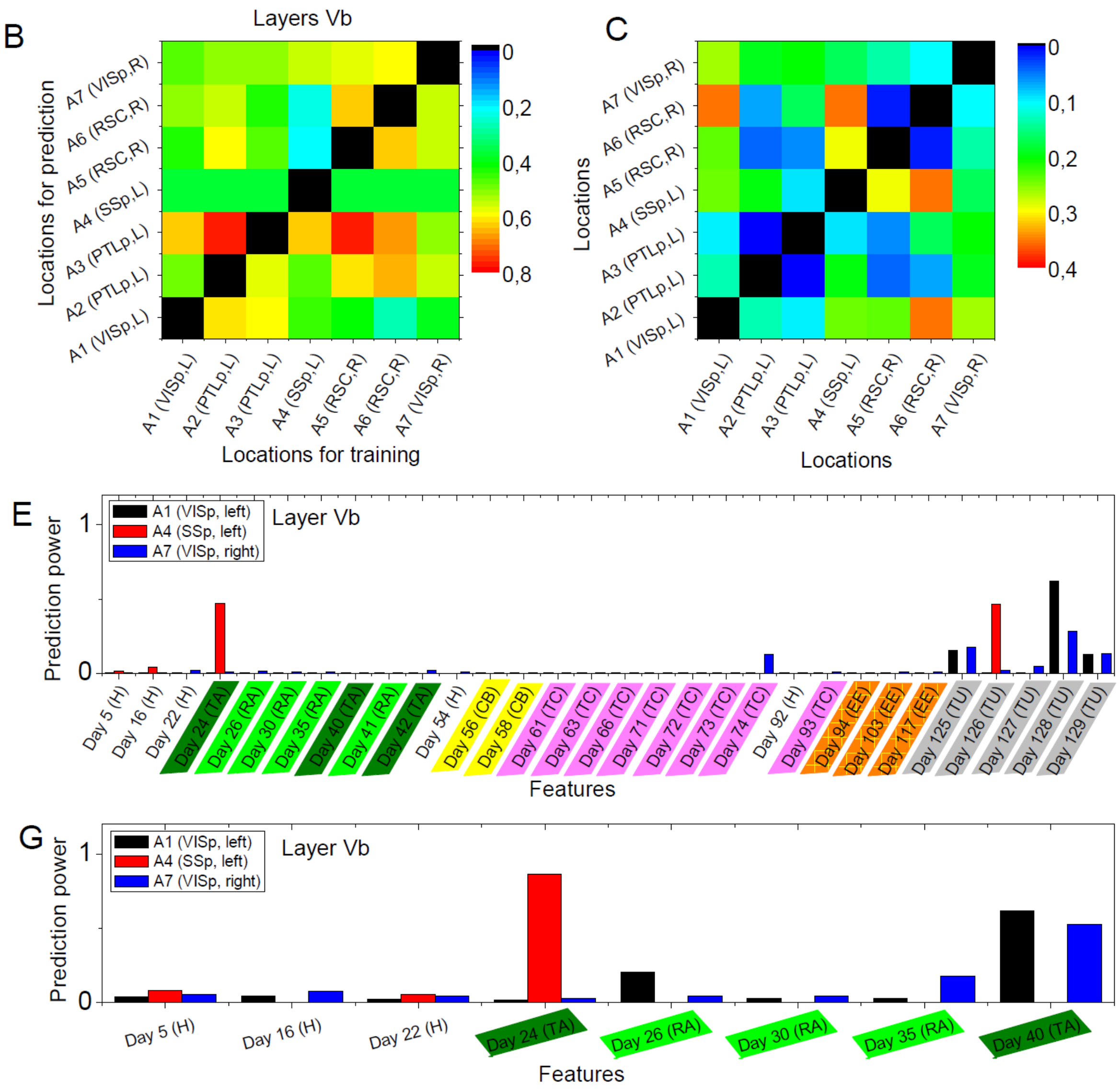

Figure S2 Cross-location prediction performance $\tilde{M}_{\kappa\Lambda}$ in layer Vb (B), and its relative difference M_s from layers II/III (C), by using the data from animal M_a. Diagonal elements do not have values. (E) Prediction power distributions of the layer Vb model trained in cortical location A1 (left VISp), A4 (left SSp), and A7 (right VISp), when the neural activities on Day 130 (Tunnel) was used as the target and all previous days in the data set as the features. (G) The prediction power distributions of layer the layer Vb model when the neural activities on Day 41 (Retrieval A) was used as the target and 8 previous days in the data set as the features. Abbreviations – H: Home cage; TA: Training A; RA: Retrieval A; CB: Context B; TC: Training C; EE: Enriched environment; and TU: Tunnel. This figure shows the results of layer Vb corresponding to those of layer V in Figure 2.

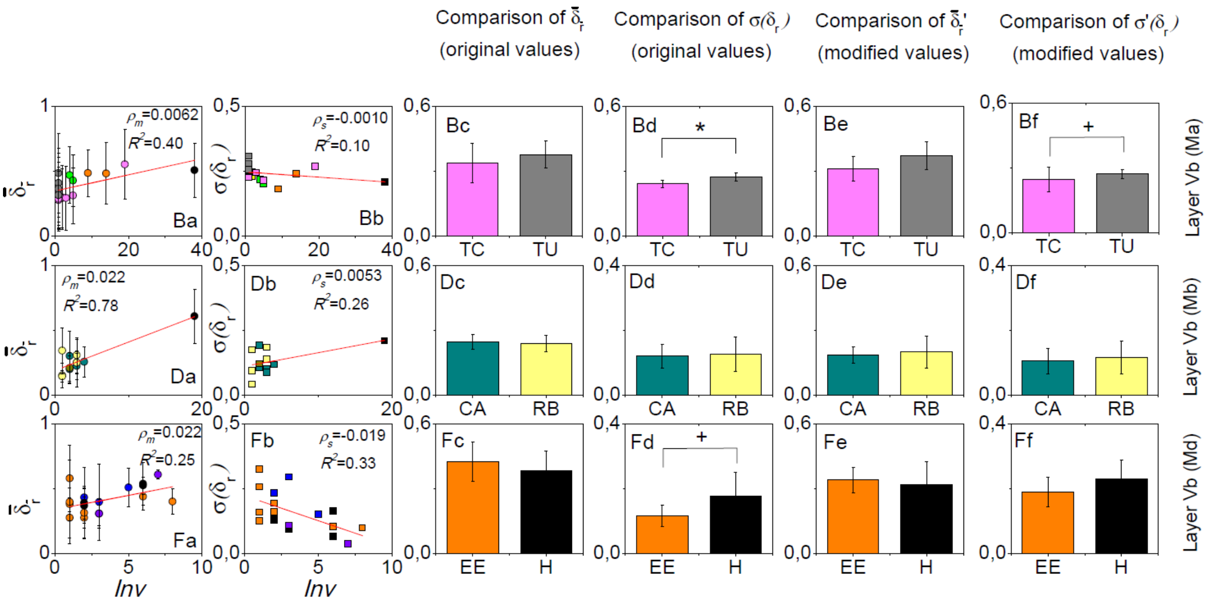

*Figure S3 Results of the environment-repeat prediction in layer Vb. The first column is the dependence of $\bar{\delta}r$ on the environment-repeat interval Inv, where error bars in fact indicate the standard deviation $\sigma(\delta r)$, and the red lines are the linear fitting results. The second column shows the dependence of $\sigma(\delta r)$ on Inv. The third to the sixth columns show the comparisons of $\bar{\delta}r$ and $\sigma(\delta r)$ with the original and modified data, respectively, between the most often repeated environments that each mouse experienced. Colours in the figure are used to discriminate environment types. +: $p<0.1$ and *: $p<0.05$. This figure shows the results of layer Vb corresponding to those of layer V in Figure 3.*
